# The Institutional Library

**Published:** 1914-07-11

**Authors:** 


### THE INSTITUTIONAL LIBRARY.

### A Medical Classic.

Tropical Diseases. By Sir Patrick Manson, G.C.M.G., M.D., F.R.C.P. Fifth Edition. (London: Cassell & Co. 1914. Pp. 937. Price 12s. 6d. net.)

It has been said that in the dwelling-place of every British official in tropical Africa there are two volumes that are invariably found: the Army and Navy Stores catalogue and Rowland Ward's "Records of Big Game." In the case of the medical officer, a third takes precedence of both these, and that is Manson's "Tropical Medicine." Counting in reprints, which have frequently contained added matter, this is the fifteenth issue of this standard text-book, though it is only the fifth to be dignified with the style and title of a separate "edition." Inasmuch as its first appearance was made just sixteen years ago, the immense popularity of the book and its dissemination throughout the English-speaking world are obvious. For that popularity we prophesy a fresh lease of life as the outcome of this fifth edition. Since the fourth one tropical medicine has forged rapidly ahead, and all the new discoveries are incorporated in the new edition, which in consequence is steadily increasing in bulk. For example, the discovery that Kala-azar, especially the infantile form, is not infrequent along the Mediterranean littoral, and also in many tropical districts not previously suspected, is one of the important researches of late years; and so is the revelation of peculiar forms of dermal leishmaniasis in South America, and of the intimate association of these diseases with the dog. The biological evolution of trypanosomes within their intermediate hosts, the tsetse flies, is another finding of great importance, since formerly a mere mechanical transmission of the infecting parasites was postulated. Then a new form of trypanosomiasis has come to light in South America, transmitted by an insect differing from the African biting flies; sand-fly fever of a three-day type has been proved to exist in certain sub-tropical and temperate regions; the causation of beri-beri has been investigated, and probably established; and scores of other instances could be quoted. In a word, Sir Patrick's text-book easily maintains the leading position which it gained on its first publication, and is a book of which British medicine may well be proud. A minor but equally praiseworthy point is the moderate price at which it is published.

### A Series from the Hospital Necker.

Manuel de Cystoscopie. Par E. Papin. (Paris: F. Gittler, 16 Rue Dauphine. 1914. Pp. 326. Price 15 fr.)

Cystoscopy has won such a series of victories in the campaign against urinary diseases that its place in the sun must be fully conceded. Many of the most striking of the developments of cystoscopic technique have seen the light in the last few years, and the applications of the method are constantly extending. It need occasion no surprise, therefore, that the time has come when the whole of a textbook is devoted purely to descriptions of cystoscopic technique and the indications for its employment. Dr. Papin has not only filled 326 large pages, but he has actually economised space by that simple and perspicuous style of writing which French scientists so often possess. He omits nothing, yet wastes no space on pretentious verbosity: he sticks closely to business, and every page is worth reading.

Quite lately there has been some controversy in the *Lancet* amongst London urologists as to the uses of diathermy for growths of the bladder, compared with other methods, especially resection. Dr. Papin sets forth his own views on the indications for intravesical methods of access as follows: For small growths of all kinds there is little to choose between diathermy, resection, and cauterisation. For pedunculated tumours up to a moderate size diathermy is dangerous; various methods of cystoscopic destruction by snare and electricity are recommended. For multiple growths, papillomatous or otherwise, diathermy is strongly advised. Recurrences of tumours removed by resection should also be dealt with intravesically, and so should periureteral growths. He sums up by stating that for growths which are apparently benign the cystoscopic method has been gaining ground (at the expense of operative measures), but that for malignant growths it has hitherto proved useless.

One of the marked features of the book is the absence of those beautiful coloured plates of cystoscopic objects with which most textbooks are replete. Instead, there are line drawings, somewhat roughly executed, in abundance. As a matter of fact, it is quite astonishing to note how little the elaborate and expensive coloured plates are missed. Considering, however, the inexpensive format of the book and its paper cover, a lower price than fifteen francs might have resulted in its reaching a wider circle of readers. Otherwise, we have nothing but praise for a volume which is a credit to the author and to the Hospital Necker, where he is *chef de clinique des voies urinaires*.

Physiologie Normale et Pathologique des Reins. Par L. Ambard. (Paris: F. Gittler, 16 Rue Dauphine. 1914. Pp. 329. Price 15 fr.)

The modern urologist depends so greatly for his diagnosis and treatment upon the assistance with which the physiologist and the pathologist can supply him that a separate volume summing up all that is now known of the functions of the normal and the abnormal kidney is bound to be of great value, both for the instruction of those taking up the study of urology and as a reference book for those who are actively practising it. Dr. Ambard's work will be found a veritable cyclopædia of urinary functions, necessarily somewhat abstruse in parts, but admirably fulfilling the aims expressed in the title of it.

Notions Pratiques d'Electrothérapie appliquée à l'Urologie. Par Dr. D. Courtade. (Paris: F. Gittler, 16 Rue Dauphine. 1914. Pp. 212. Price 10 fr.)

Dr. Courtade is an enthusiastic electrotherapist, and has evidently acquired much first-hand knowledge of the possibilities of electrotherapy in urology. For various reasons, however, his exposition of the subject fails to communicate an equal enthusiasm to his readers. At the same time, his book contains an enormous amount of information, since he starts *ab initio*, as it were, and treats of the subject of electricity from the most elementary up to the most advanced planes. It is in the applications of electricity to urinary therapeutics that the author seems to us to claim rather too much. This, however, remains for time to show; possibly it will prove that electricity can do even more for these diseases than Dr. Courtade now contends it can.

THE SOCIAL DISEASE, AND HOW TO FIGHT IT. By LOUISE CREIGHTON. (Longmans, Green. Price 1s. net.)

IN this little book, which its distinguished authoress herself styles a rejoinder, an effort is made to counteract that intense and inveterate hostility to men which is the keynote of a book lately issued by Miss C. Pankhurst. The latter publication contains a discussion of sexual matters, and especially of the venereal diseases, of extraordinary frankness. Exaggerated in tone, it is packed with the grossest over-statements and mis-statements of male perfidy and infectivity. The impression given is that ninety-nine men out of a hundred are ravening wolves, perpetually on the alert to ruin all women, and soaked in the poisons of venereal diseases. The extraordinarily bitter, narrow views of the aforesaid writer find no echo in this present volume. The widow of the late Bishop of London does, certainly, face plain facts as courageously as Miss Pankhurst, but she takes a great deal more trouble to make sure of them before rushing into print. She also sees—what Miss Pankhurst is clearly incapable of doing—that there are two sides to the sex controversy, and that insistence on one side only is not likely to convince those who happen by the accident of birth to be well acquainted with aspects of the other which are ignored or overlooked by the more vehement advocates of modern "feminism." We would not be thought to endorse everything that Mrs. Creighton says, but we do heartily welcome this appeal to reason and common sense after the frothy hysterics of the inaccurate Miss Pankhurst.

POCKET FORMULARY FOR THE TREATMENT OF DISEASE IN CHILDREN. By LUDWIG FREYBERGER, J.P., M.R.C.P. Fourth Edition. (London: Wm. Heinemann. 1914. Pp. 260. Price 7s. 6d.)

THE fact that this work has reached a fourth edition, and this in spite of the very considerable unpopularity of its author with a large section of the medical profession, is evidence of its merit and value. After looking carefully through it, we can add our testimony to the same effect. The "Pocket Formulary" is not, and we suppose Dr. Freyberger would not claim that it is, a work of genius or an exhibition of great clinical acumen. It is simply a compilation which has required very great industry and a discriminating judgment. No one else having taken the necessary pains, or made a happier selection, the author is entitled to the credit and the cash as well. His book has made a place for itself, and there is no difficulty in understanding the reason.

